# Immunological landscape of nanotechnology-based depression research: a bibliometric analysis of neuroinflammation and immune modulation

**DOI:** 10.3389/fimmu.2026.1791933

**Published:** 2026-05-01

**Authors:** Zi-yi Guo, Jian-hua Zou, Yan-chen Feng, Zhuo-yu Ren, Wen-bin Wang, Juan-juan Feng, Jie Zhao

**Affiliations:** 1 National Engineering Laboratory for Internet Medical Systems and Applications, The First Affiliated Hospital of Zhengzhou University, Zhengzhou, Henan, China; 2 Department of Rehabilitation Medicine, The First Affiliated Hospital of Zhengzhou University, Zhengzhou, Henan, China; 3 School of Basic Medical Sciences, Heilongjiang University of Chinese Medicine, Harbin, Heilongjiang, China; 4 Department of Anesthesiology, Pain and Perioperative Medicine, The First Affiliated Hospital of Zhengzhou University, Zhengzhou, Henan, China; 5 Department of Pharmacy, The First Affiliated Hospital of Zhengzhou University, Zhengzhou, Henan, China

**Keywords:** bibliometric, depression, immunity, microglia, nano, neuroinflammation

## Abstract

**Purpose:**

Immune dysregulation and neuroinflammation have been increasingly implicated in depression, and nanotechnology-based approaches are being actively explored for targeted delivery and immunomodulation. However, a systematic, immune-oriented bibliometric mapping of research at the intersection of nanotechnology and depression remains limited. This study aims to characterize the publication landscape, collaboration patterns, and thematic evolution of this field using bibliometric evidence.

**Methods:**

Relevant publications (up to December 8, 2025) were retrieved from the Web of Science Core Collection and Scopus. Bibliometric visualization and network analyses were performed using Bibliometrix, VOSviewer, and CiteSpace to examine publication trends, global collaborations, core journals, and immune-related research themes.

**Results:**

A total of 591 publications were included, showing marked growth after 2020. China and the United States were leading contributors in publication output and citation impact. Keyword co-occurrence, burst detection, and co-citation analyses indicated a thematic evolution from earlier emphases on neurotransmitter-related delivery/stabilization toward increasing attention to neuroinflammation- and immune-modulation–related topics. Recent hotspots and bursts were frequently associated with microglial biology/polarization, oxidative stress, and pro-inflammatory cytokines.

**Conclusion:**

This bibliometric study provides a descriptive overview of how research attention within nanotechnology and depression has evolved, with increasing prominence of immune- and neuroinflammation-related themes. These trend-based findings may help prioritize directions for future experimental and translational investigations.

## 1 Introduction

Depression is among the most prevalent and disabling neuropsychiatric disorders worldwide and remains a leading contributor to the global burden of disease and years lived with disability (1). Clinically, it is a highly heterogeneous condition characterized by persistent low mood, anhedonia, and a wide range of psychological and somatic symptoms (2–4). These manifestations substantially impair quality of life and impose significant emotional, physical, and economic burdens on individuals, families, and healthcare systems (5). The pathogenesis of depression is multifactorial, involving genetic susceptibility, dysregulation of monoaminergic neurotransmission, hypothalamic–pituitary–adrenal axis dysfunction, neuroinflammation, oxidative stress, and impaired neuroplasticity (6–9). The growing recognition of immunological contributions to depression has progressively influenced contemporary disease models and therapeutic paradigms (10).

Neuroinflammation and aberrant immune activation are now widely implicated in the onset, progression, and treatment resistance of depression (11). Dysregulated microglial activation, altered cytokine signaling, and disrupted peripheral–central immune communication can profoundly influence synaptic function, neuronal integrity, and neuroplasticity, thereby contributing to depressive phenotypes (12–14). However, the multifactorial and interconnected nature of these immune and neural mechanisms presents a major challenge for therapeutic development. Current antidepressant strategies remain largely rooted in monoaminergic theories, which capture only a fraction of the underlying biological complexity and are associated with delayed onset of action, heterogeneous treatment responses, and limited long-term efficacy (15). These limitations underscore an urgent need for innovative approaches capable of simultaneously targeting immune, inflammatory, and neural pathways.

In this context, nanotechnology has emerged as a versatile and promising platform for addressing the multifaceted pathogenesis of depression. The unique physicochemical profile of nanomaterials—characterized by tunable size, tailorable surface chemistry, and robust drug-loading—enables them to facilitate transport across the blood–brain barrier and potentially interact with neural immune cells, thereby optimizing pharmacokinetics and therapeutic targeting (16–18). Nanocarrier-based systems can enhance central nervous system bioavailability while minimizing systemic adverse effects. Importantly, nanoscale platforms are increasingly recognized not merely as passive delivery vehicles but as active therapeutic agents capable of modulating neuroinflammatory, oxidative stress, and immunoregulatory pathways directly implicated in depression pathophysiology (19–21). In parallel, nanotechnology-enabled diagnostic and imaging strategies have facilitated sensitive detection and real-time monitoring of immune-related processes within the brain (22, 23). Corresponding with these technological advances, research at the intersection of nanotechnology and depression has expanded rapidly over the past two decades, giving rise to a distinct interdisciplinary field integrating neuroscience, psychiatry, immunology, materials science, pharmacology, and biomedical engineering.

Bibliometric analysis provides a quantitative and visualized approach for mapping scientific development through the assessment of publication outputs, collaboration networks, citation structures, and keyword co-occurrence patterns. From an immunological perspective, such an approach is particularly valuable for elucidating the transition from broadly anti-inflammatory nanocarrier strategies toward precision immunomodulatory nanotherapies. Therefore, the present study aims to systematically map the knowledge structure and thematic evolution of nanotechnology-related depression research, with a specific focus on immune and neuroinflammatory dimensions. By delineating the immunological footprint of this interdisciplinary field, this work aims to inform future research directions and the clinical translation of immunomodulatory nanomedicine in depression.

## 2 Methods

### 2.1 Literature sources and search strategy

A comprehensive literature search was conducted using the Web of Science Core Collection (WoSCC) and Scopus databases to achieve broad and representative coverage of research on nanotechnology applications in depression. These databases were selected due to their extensive indexing of peer-reviewed literature, standardized bibliographic records, and widespread use in bibliometric studies. The search strategy combined two concept domains: depression and nanotechnology. Depression-related search terms included: “depression”, “major depressive disorder”, “persistent depressive disorder”, “seasonal affective disorder”, and “postnatal depression”. Nanotechnology-related studies were identified using the wildcard term “nano*”, which captures a broad spectrum of relevant concepts, including nanoparticles, nanocarriers, nanomedicine, and nanoscale systems. The two domains were combined using Boolean operators; an example search string in WoSCC was: TS= (“depression” OR “major depressive disorder” OR “persistent depressive disorder” OR “seasonal affective disorder” OR “postnatal depression”) AND TS=(nano*). No lower date restriction was applied, and all publications available up to December 8, 2025 were included. Only articles published in English were considered to ensure data consistency and cross-database comparability. Detailed search strategies for each database are provided in **Supplementary Table 1**.

### 2.2 Data integration, inclusion, and exclusion criteria

Records retrieved from WoSCC and Scopus were consolidated and managed in Microsoft Excel 2021. Duplicate records were identified by cross-checking titles, author names, publication years, and digital object identifiers (DOIs). In cases of uncertainty, ORCID identifiers and manual verification were used to ensure accurate author identification. Publications classified as meeting abstracts, editorials, letters, conference proceedings, book chapters, corrections, and retracted articles were excluded. Two independent reviewers screened titles and abstracts for relevance, with any disagreements resolved through discussion with a third reviewer. Full-text evaluation was performed when the title and abstract were insufficient to determine eligibility. To enhance data consistency and analytical accuracy, bibliographic records underwent systematic preprocessing, including: (i) standardization of author names and institutional affiliations; (ii) normalization of journal titles; and (iii) curation of author keywords by merging synonymous terms and removing non-informative or overly generic expressions. Records with missing essential bibliographic information were also excluded. Inter-reviewer agreement was monitored throughout the screening process.

### 2.3 Bibliometric analysis and visualization

Bibliometric analyses and visualizations were conducted using Bibliometrix (R package v4.1, RStudio v4.3), VOSviewer (v1.6.19), and CiteSpace (v6.2). Bibliometrix was applied for descriptive statistics, including annual publication trends, citation performance, and journal distribution. VOSviewer was used to construct co-authorship networks at the country, institution, and author levels, where nodes represent entities and links denote co-authored relationships. Keyword co-occurrence analysis was performed to identify research hotspots and thematic structures. CiteSpace was employed for keyword burst detection to capture emerging topics and temporal shifts in research attention. In addition, reference co-citation analysis and timeline visualizations were conducted in CiteSpace to map the intellectual base and the temporal evolution of major research themes. Finally, trend inference was derived from historical keyword occurrence patterns to indicate potential future directions. To identify core authors, Price’s Law was applied using the commonly adopted bibliometric formulation: M = 0.749×√Nmax. where *N*max denotes the maximum number of publications by a single author in the dataset, and *M* represents the minimum publication count for core authors. In this dataset, M was calculated as 3.

## 3 Results

**Figure 1:**
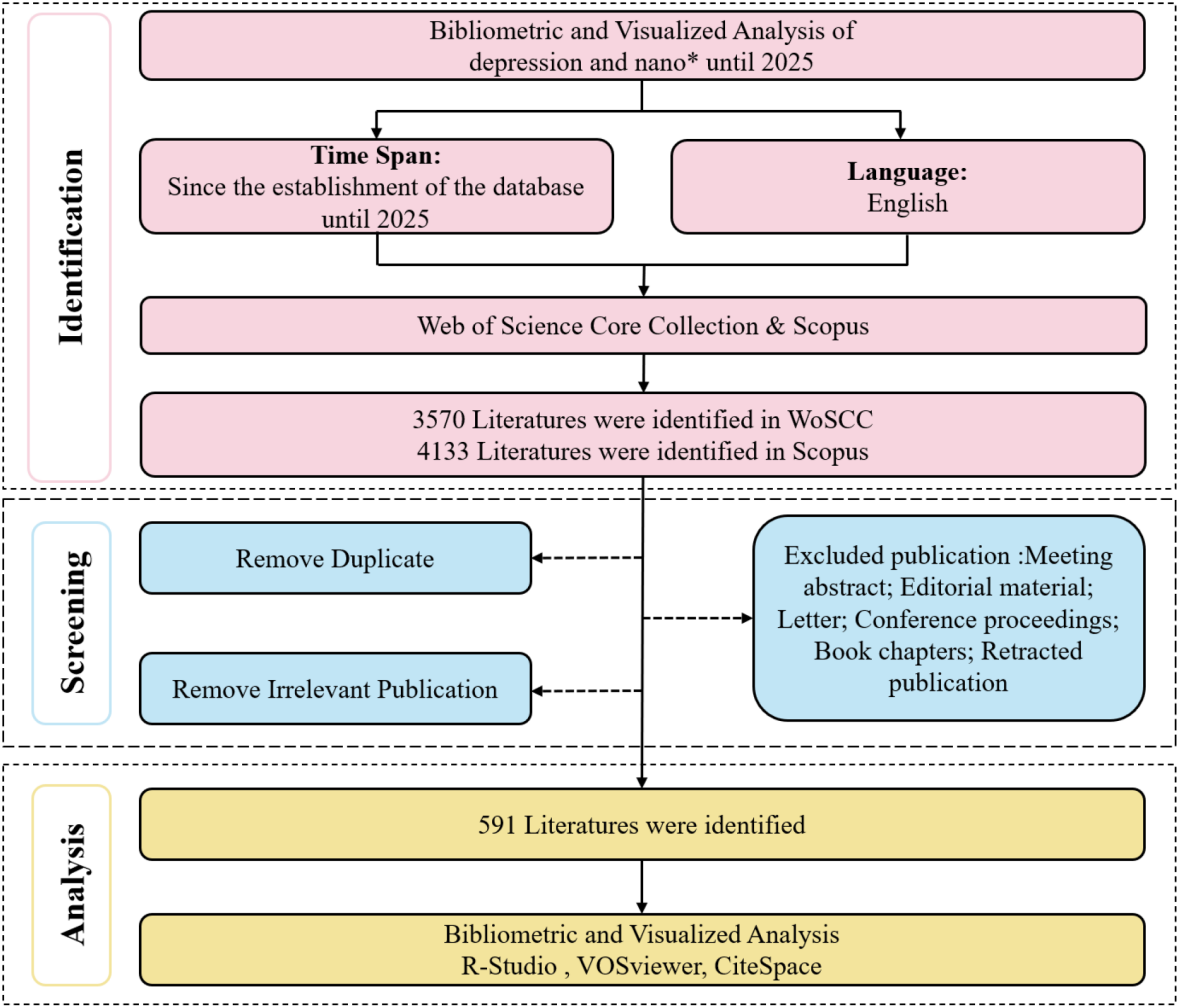
Flowchart of data collection for included studies.

A total of 7,916 records were initially retrieved from the Web of Science Core Collection and Scopus databases, covering the period from database inception to December 8, 2025. After removing duplicate records and screening titles and abstracts to exclude irrelevant publications, 591 articles were retained for subsequent bibliometric analysis (**Figure 1**).

### 3.1 Trend in publications

**Figure 2:**
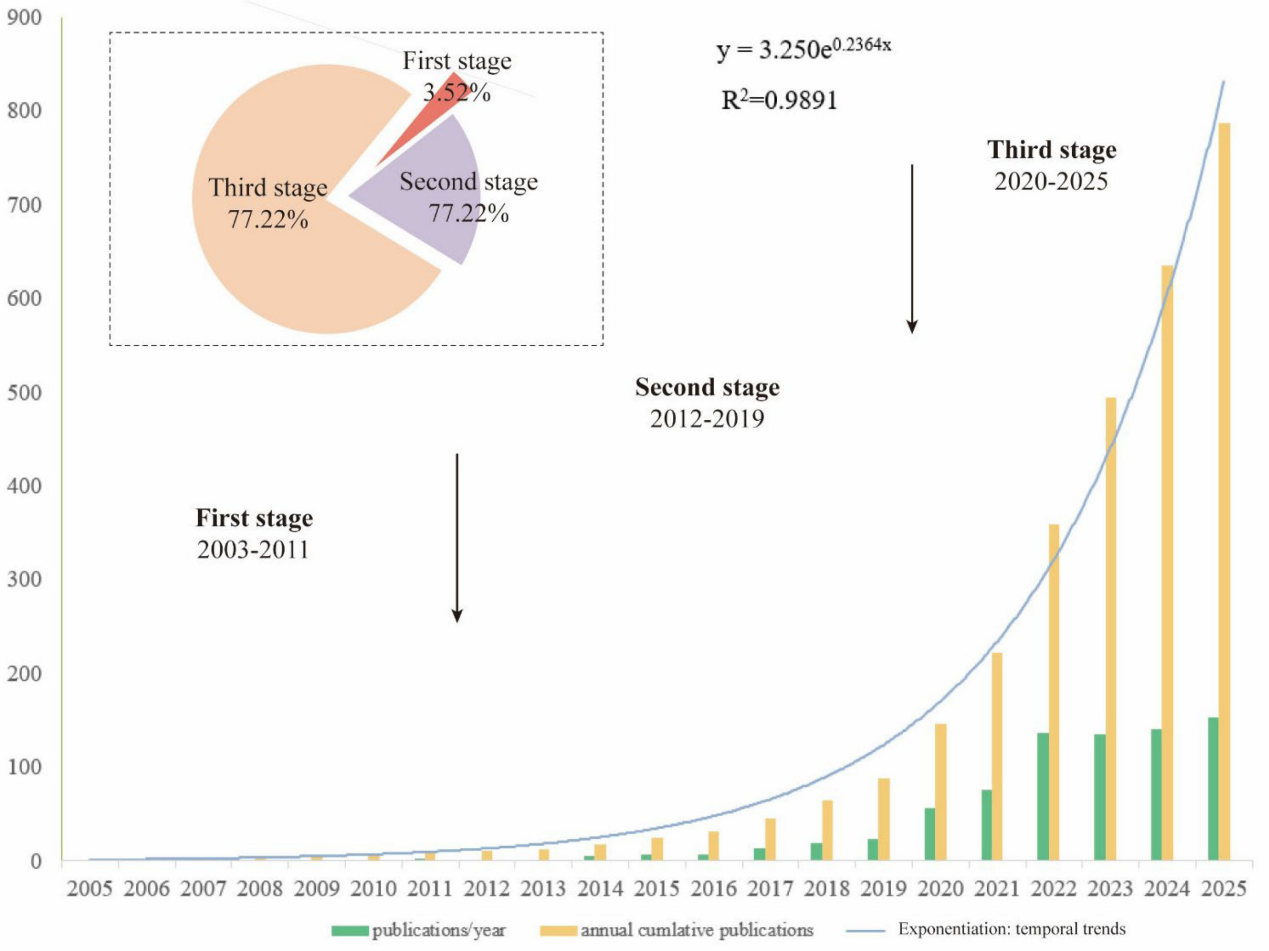
The distribution of publication dates.

Annual publication output was analyzed to examine temporal trends in nano–depression research (**Figure 2**). The results revealed a clear three-phase developmental trajectory. The first phase (2003–2011) was characterized by minimal research output, representing a dormant period in the field. The second phase (2012–2019) showed gradual growth, with a total of 115 publications and fewer than 30 articles published per year. The third phase began in 2020 and was marked by a pronounced surge in publication activity, accounting for 461 articles. Overall, the cumulative publication trend followed an exponential growth pattern (R² = 0.989), indicating sustained and accelerating research interest in this interdisciplinary field.

### 3.2 Country-level collaboration and co-authorship network

**Figure 3:**
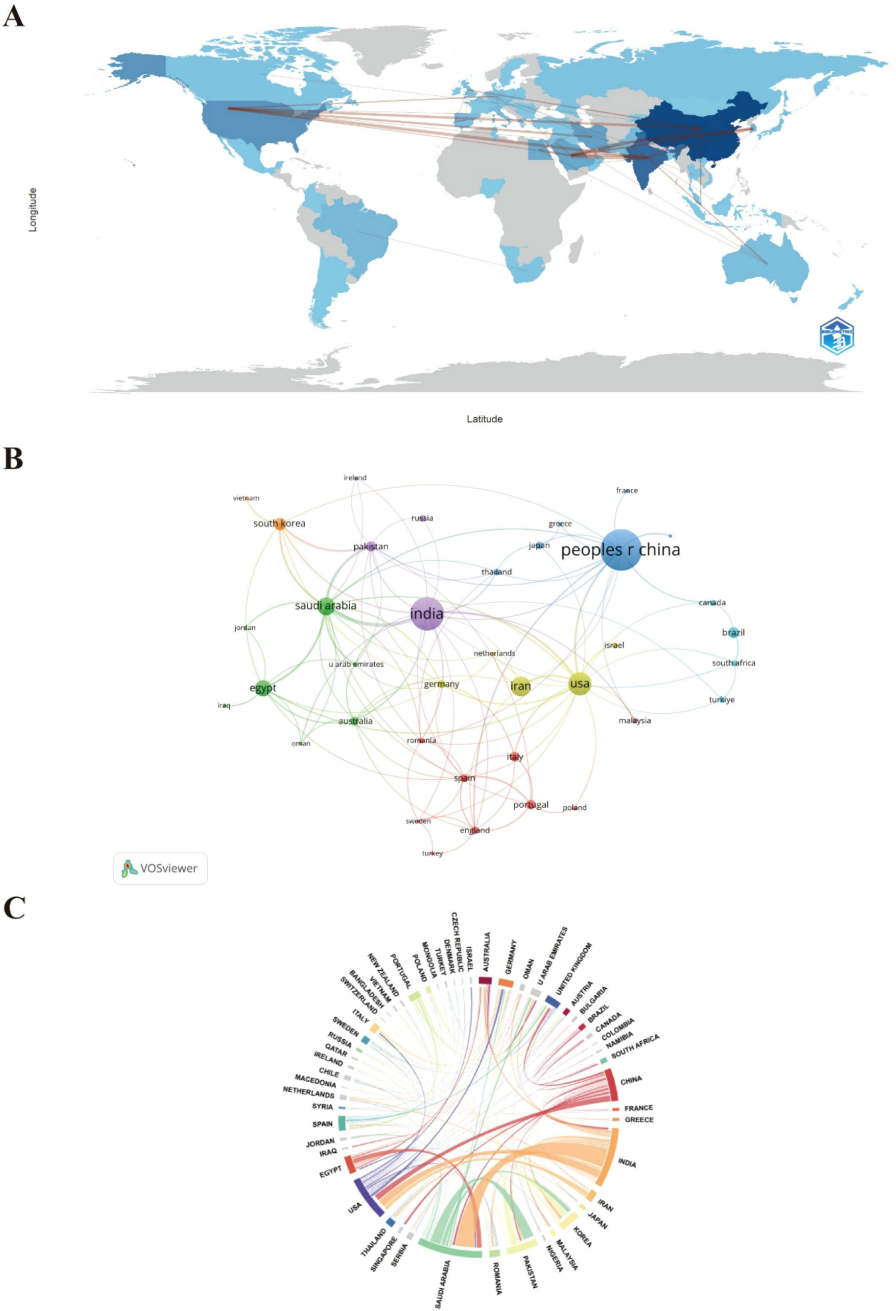
Co-authorship network among countries: **(A)** global collaboration. **(B)** International research collaboration network. **(C)** Chord diagram of international collaborations.

Country-level co-authorship analysis was conducted to examine global collaboration patterns in nano–depression research (**Figures 3A–C**). China, the United States, and India emerged as the most active contributors, although their roles differed in terms of productivity, citation impact, and collaborative engagement. China produced the highest number of publications (n = 170), accounting for 35.87% of the total output. India ranked second in publication volume (n = 113) and demonstrated the highest level of international collaboration, maintaining research partnerships with 19 countries, most notably Saudi Arabia. The United States collaborated with 17 countries and exhibited the highest total and average citation counts, reflecting its strong research impact despite a comparatively lower publication volume. In contrast, several countries, including Denmark, Singapore, Vietnam, Egypt, and the Czech Republic, maintained collaborations with only a single partner. These patterns indicate substantial heterogeneity in international collaboration intensity across countries (**Table 1**).

**Table 1:** The top 10 productive countries.

Country	N	Citations	Average citations	%
China	170	2591	15.24	35.87
India	113	1863	16.49	23.84
Iran	47	626	13.32	9.91
USA	43	4242	98.65	9.07
Egypt	34	449	13.21	7.17
Brazil	19	202	10.63	4.01
South Korea	17	428	25.18	3.59
Portugal	12	451	37.58	2.53
Pakistan	11	321	29.19	2.32
Saudi Arabia	8	117	14.63	1.69

### 3.3 Core authors and author collaboration networks

**Figure 4:**
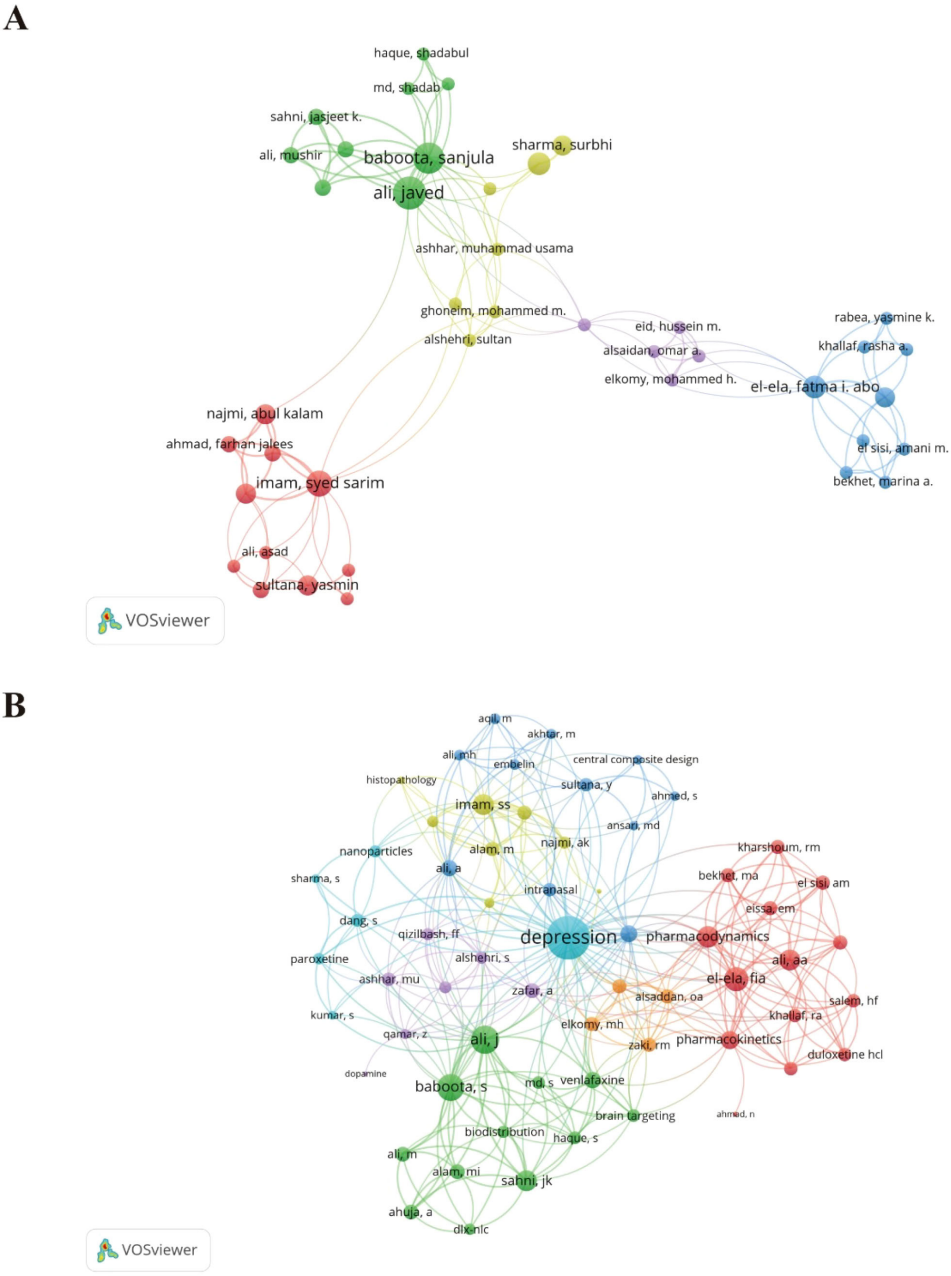
Co-authorship network among core authors: **(A)** core authors collaboration, **(B)** core authors’ focus themes.

Author productivity was analyzed to identify core contributors to the field. According to Price’s Law, authors publishing at least M = 3 articles were defined as core authors. Based on this criterion, 308 core authors were identified, indicating a relatively stable research community. Co-authorship network analysis revealed that only 41 core authors formed interconnected collaboration networks, which were organized into five distinct clusters (**Figure 4A**). These clusters reflected thematic specialization within the field. As illustrated in **Figure 4B**, Cluster 2, represented by Ali Javed and Baboota Sanjula, primarily focused on brain-targeted drug delivery, venlafaxine formulations, and biodistribution studies. Cluster 1, centered around Sultana Yasmin, and Cluster 4, led by Dang Shweta, predominantly investigated nanoparticle-based antidepressant delivery and intranasal administration strategies. Overall, the author collaboration network exhibited limited connectivity, suggesting that research efforts remain relatively fragmented.

### 3.4 Leading institutions

Institutional analysis identified the top ten contributing institutions in nano–depression research (**Table 2**). Jamia Hamdard University (India) ranked first with 23 publications, followed by King Saud University (Saudi Arabia, n = 17) and Tehran University of Medical Sciences (Iran, n = 15). Three of the top ten institutions were located in China, two in Iran, and the remaining institutions were distributed across India, Saudi Arabia, Egypt, Portugal, and Russia. Collectively, these institutions produced 135 publications, accounting for 22.61% of the total output, and accumulated 3,075 citations. Network visualization revealed relatively dense collaborative links among leading institutions, highlighting the role of regional research hubs in advancing nano–depression studies.

**Table 2:** Top 10 institutions of high contribution.

Institution	Country	Documents	Total citations	Average citations
Jamia Hamdard University	India	23	876	38.09
King Saud University	Saudi Arabia	17	328	19.29
University of Tehran for Medical Sciences	Iran	15	305	20.33
Chinese Academy of Sciences	China	14	276	19.71
Islamic Azad University	Iran	12	246	20.50
Lanzhou University	China	12	221	18.42
Shanghai Jiao Tong University	China	11	115	10.45
Cairo University	Egypt	11	189	17.19
Universidade de Coimbra	Portugal	11	410	37.27
Dmitry Rogachev National Medical Research Centre of Pediatric Hematology	Russia	9	109	12.11

### 3.5 High‐contributing journals

**Figure 5:**
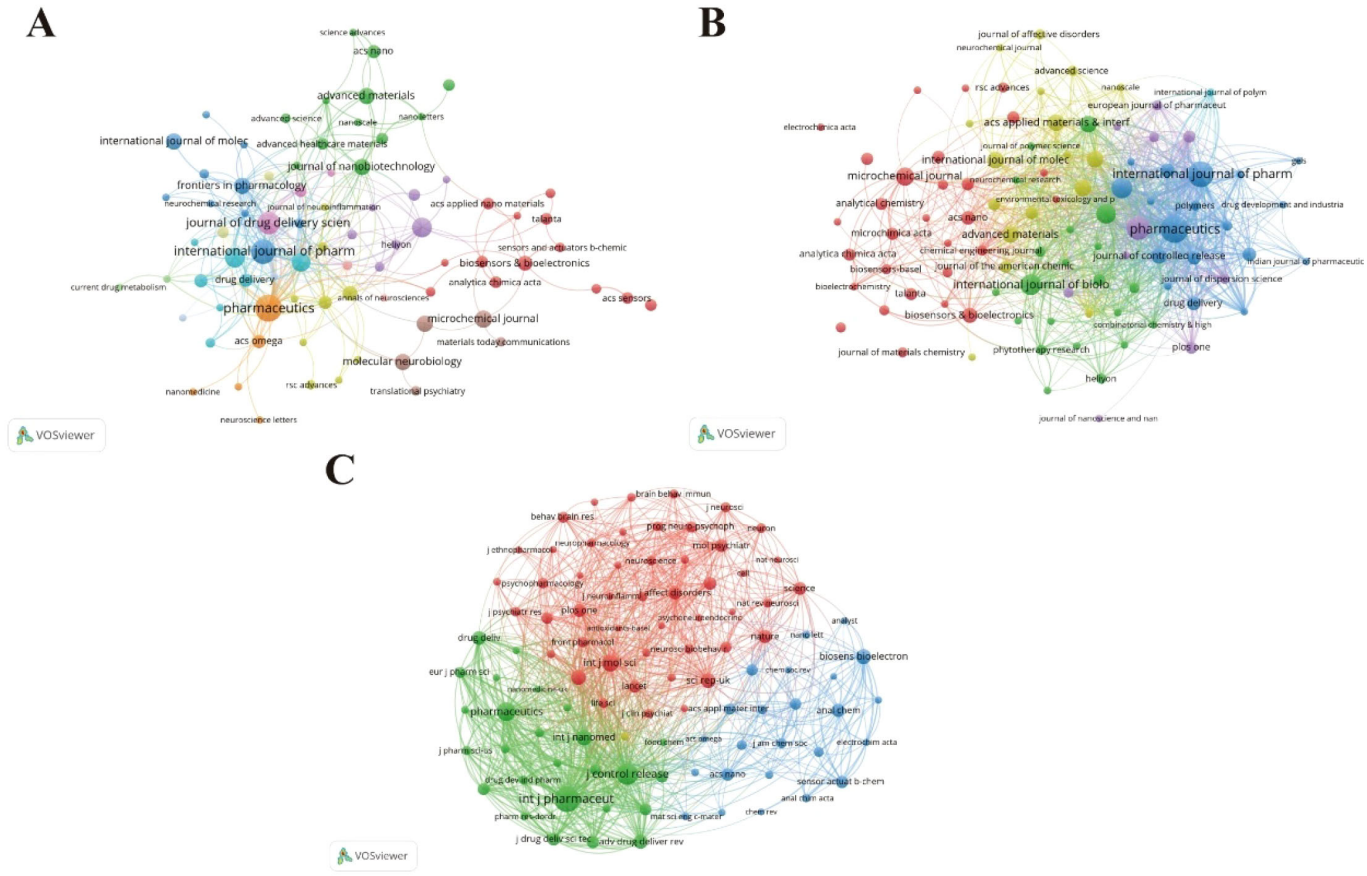
Journal relationship analysis: **(A)** journal citation analysis; **(B)** journal coupling analysis; **(C)** journal co-citation analysis.

Journal-level analyses were performed to characterize the influence, research structure, and intellectual foundations of nano–depression research, including journal citation, bibliographic coupling, and co-citation analyses (**Figures 5A–C**). The top ten journals collectively published 100 articles, accounting for 16.75% of the total output, indicating a moderate concentration of research activity within a limited number of journals. Pharmaceutics published the largest number of articles (n = 16) and received the highest total citations (n = 367). The International Journal of Biological Macromolecules exhibited the highest average citations per article (31.30), reflecting strong research influence (**Table 3**). Journal citation analysis showed that a small number of high-impact journals accounted for a disproportionate share of total citations. These journals primarily spanned nanotechnology, pharmacology, biomedical engineering, and neuroscience, underscoring the interdisciplinary nature of the field. Bibliographic coupling analysis revealed several distinct journal clusters sharing similar reference patterns, representing the contemporary research structure. The largest clusters were dominated by journals focusing on nanomedicine and drug delivery. In contrast, journal co-citation analysis revealed more consolidated and stable clusters, indicating a relatively consistent intellectual foundation rooted in neuroscience, pharmacology, and nanotechnology.

**Table 3:** Top 10 citing sources.

Journal	N	Total citations	Average citations	JCR2025	Discipline
Pharmaceutics	16	367	22.94	Q1	Medicine, Pharmacy
International Journal of Pharmaceutics	14	310	22.14	Q1	Medicine, Pharmacy
Journal of Drug Delivery Science and Technology	12	124	10.33	Q1	Medicine, Pharmacy
International Journal of Biological Macromolecules	10	313	31.30	Q1	Biochemistry and Molecular Biology, Applied Chemistry, Polymer Science
Pharmaceuticals	10	94	9.40	Q1	Medicine, Pharmacy
International Journal of Nanomedicine	9	204	22.67	Q1	Pharmacy, Nanotechnology
Microchemical Journal	8	40	5.00	Q1	Chemistry, Analytical Chemistry
Journal of Nanobiotechnology	7	131	18.71	Q1	Bioengineering and Applied Microbiology, Nanotechnology
Frontiers in Pharmacology	7	59	8.43	Q1	Pharmacy
Molecular Neurobiology	7	56	8.00	Q1	Medicine, Neuroscience

### 3.6 Highly cited references

Analysis of highly cited references provided insights into the intellectual foundations of nano–depression research (**Table 4**). The most frequently cited study was published by Lai et al. (2003), which introduced a mesoporous silica nanosphere-based carrier system with removable nanoparticle caps for stimuli-responsive drug release. This work established a foundational nanoplatform for controlled molecular delivery and has exerted sustained methodological influence. The second most cited publication, a review by Anand et al. (2008), systematically summarized the biological activities and therapeutic potential of curcumin and its analogues, providing a conceptual framework for subsequent nano-formulation studies. The third-ranked article by Baron et al. (2005) demonstrated neurotransmitter-induced growth of gold nanoparticles for sensitive neurochemical detection, representing an early application of nanomaterials in neuroanalytical methods. Collectively, these highly cited studies highlight the central roles of nanotechnology-enabled delivery systems, bioactive compound optimization, and innovative analytical techniques in shaping the field.

**Table 4:** Top 10 most cited references.

First author(ref.)	Country	Year	Times cited	Type	Description
Cheng-Yu Lai (24)	USA	2003	1552	Article	CdS nanocrystals serving as removable caps for an MCM-41-type mesoporous silica nanosphere-based controlled-release delivery system enable stimuli-responsive drug release.
Preetha Anand (25)	USA	2008	966	Review	How to overcome the limitations of curcumin and develop more potent derivatives or formulations.
Ronan Baron (26)	Israel	2005	358	Article	A quantitative detection method utilizing neurotransmitters to promote the generation of gold nanoparticles.
Bo Wang (27)	USA	2022	292	Article	A flexible biosensor based on an aptamer-FET array enables real-time detection of ultralow concentrations of cortisol in sweat.
Kashif Mahmood (28)	Pakistan	2015	218	Review	To overcome the challenge of curcumin’s low bioavailability, techniques such as liposome encapsulation and polymeric micelles are employed.
Daniela R. Radu (29)	USA	2004	202	Article	The polylactic acid-coated mesoporous silica fluorescence sensor specifically detects amino-containing neurotransmitters in aqueous solution.
Shadabul Haque (30)	India	2014	151	Article	Alginate nanoparticles, serving as a nose-to-brain delivery carrier for venlafaxine, significantly enhance the efficiency of drug delivery to the brain.
Katharine R. Smith (31)	USA	2014	141	Article	The psychiatric risk molecule ankyrin-G has been localized to nanoscale functional domains within synapses, opening new avenues for understanding the synaptic pathology of mental disorders.
Jahanshir Tavakolizadeh	Iran	2018	140	Review	miRNA and exosomes are expected to emerge as a novel direction for the objective diagnosis of depression.
Shadabul Haque (32)	India	2012	139	Article	Chitosan nanoparticles serve as an efficient nose-to-brain delivery system for venlafaxine, significantly enhancing the efficiency of drug delivery to the brain.

### 3.7 Research frontiers: an analysis based on author keywords

**Figure 6:**
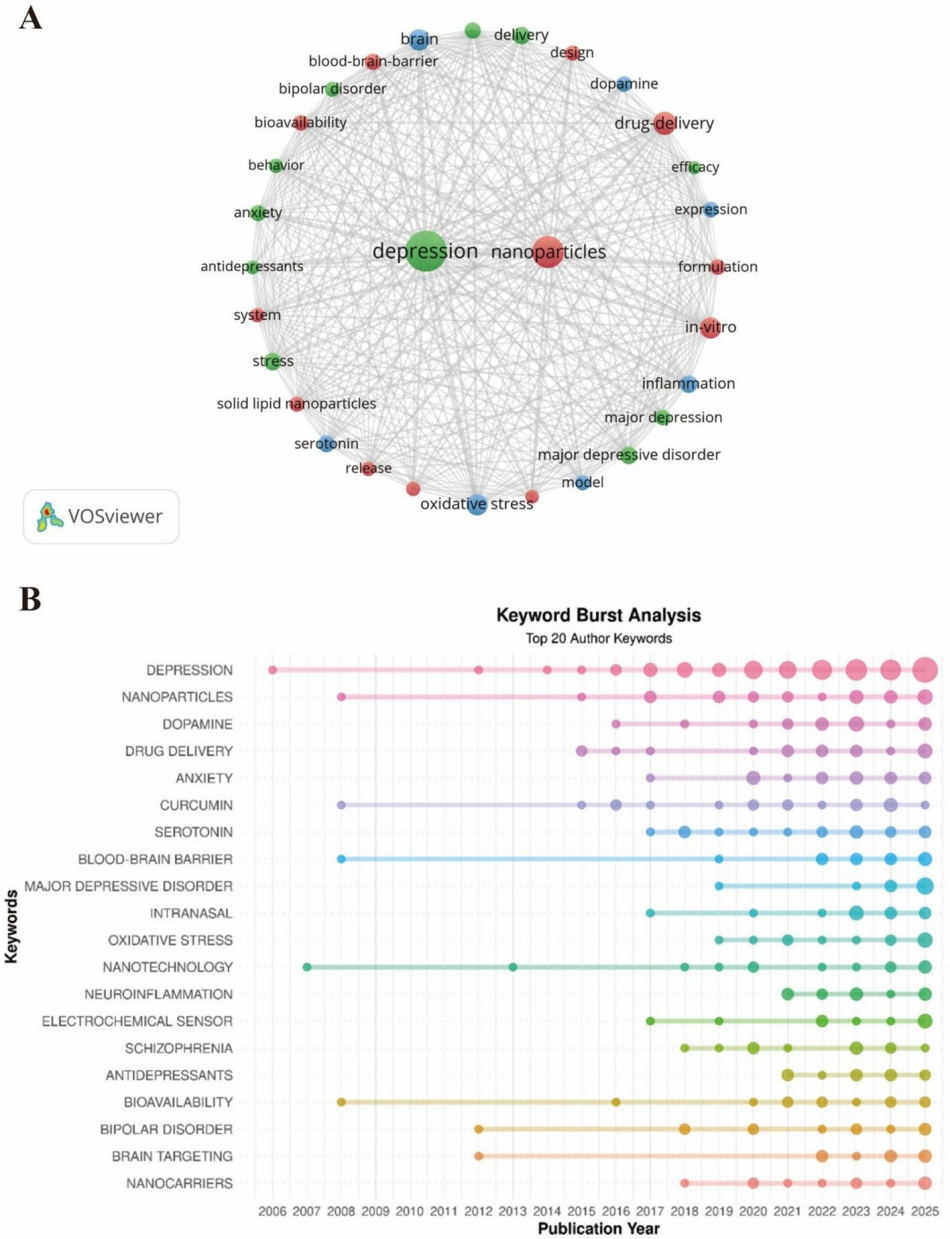
**(A)** Keyword co-occurrence network (top 30): node size, productivity; edges, co-keyword. **(B)** Temporal evolution of the top 20 most flrequent author keywords.

After merging synonymous terms and removing non-informative keywords, the top 30 author keywords were analyzed to identify research hotspots and thematic evolution (**Figure 6A**). High-frequency keywords such as “depression,” “nanoparticles,” and “drug delivery” occupied central positions in the co-occurrence network, indicating that nano-enabled delivery strategies constitute a major research focus. Mechanism-related keywords, including “oxidative stress” and “inflammation,” also appeared frequently and exhibited strong co-occurrence relationships with nanotechnology-related terms. This pattern suggests increasing integration of nano-enabled delivery systems with investigations of pathological mechanisms beyond classical neurotransmitter frameworks. Keyword burst analysis revealed a clear temporal evolution of research themes (**Figure 6B**). Early bursts were associated with neurotransmitters and basic nanotechnology concepts. During the intermediate phase, bursts related to blood–brain barrier penetration, bioavailability, and intranasal delivery became prominent. In recent years, sustained bursts were observed for keywords such as nanoparticles, nanocarriers, oxidative stress and neuroinflammation, indicating a shift toward mechanism-oriented nano-enabled therapeutic strategies.

### 3.8 Potential emerging topics for future research

**Figure 7:**
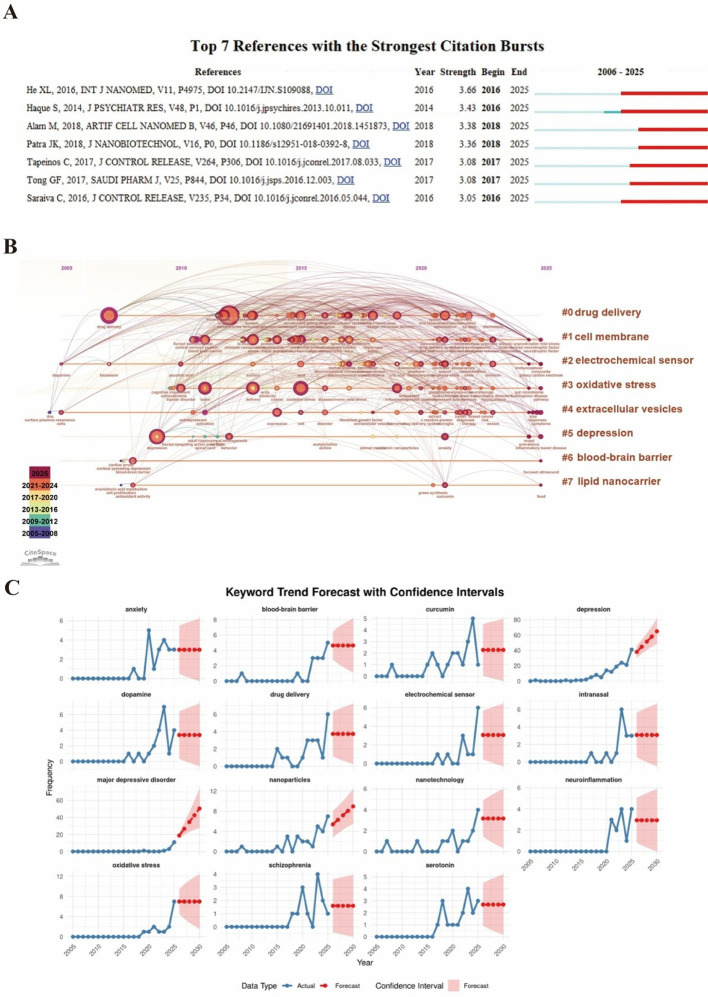
**(A)** Top 7 references with the strongest citation bursts; **(B)** timeline visualization of co-citation clusters; **(C)** keyword trend forecast with confidence intervals.

Citation burst analysis identified seven references with the strongest citation bursts between 2006 and 2025 (**Figure 7A**). All burst references emerged after 2016, reflecting intensified research activity in recent years. Among them, the study by He et al. (2016) exhibited the strongest and longest-lasting citation burst, suggesting sustained influence within the field. Co-citation cluster analysis identified seven thematic clusters (**Figure 7B**). Early clusters focused on electrochemical sensors and extracellular vesicles, whereas the largest and most prominent cluster centered on nanoparticle-based drug delivery. The most recent cluster was labeled “depression” and included emerging terms related to hippocampal function, neurogenesis, anxiety, and inflammatory bowel disease, indicating increasing attention to disease-specific neural substrates and comorbidities. Trend forecasting of the top 15 keywords suggested that “depression,” “nanoparticles,” and “major depressive disorder” are likely to exhibit continued growth in the coming years (**Figure 7C**), reflecting sustained research interest and a gradual shift toward clinically defined frameworks.

## 4 Discussion

### 4.1 Rapid growth reflects increasing integration of nanotechnology and immunological perspectives in depression research

The temporal analysis of publication output revealed a clear three-phase developmental trajectory in nanotechnology-related depression research. The prolonged dormant period prior to 2011 likely reflects early conceptual exploration and technological immaturity. Gradual growth observed between 2012 and 2019 coincided with advances in nanomaterial synthesis, drug delivery systems, and recognition of the limitations of conventional antidepressant therapies. The pronounced surge in publications after 2020 suggests expanding interdisciplinary interest encompassing both technological innovation and research attention to immune dysregulation, neuroinflammation, and oxidative stress. This growth highlights the increasing incorporation of immunological perspectives into the field, with neuroimmune regulation emerging as a prominent research theme.

From an immunological perspective, this accelerated growth coincides with increasing scholarly attention to immune-related dimensions of depression research, particularly processes such as microglial activation and cytokine imbalance, which have become more visible in the recent literature (33). Within this context, nanotechnology has attracted growing interest, likely due to its potential relevance to drug delivery, brain-targeted transport, and studies addressing immune-related aspects (34). The bibliometric pattern observed here suggests that nanotechnology is receiving increasing attention as a research approach in studies of immune-related aspects of depression, rather than serving solely as an auxiliary tool. This trend is consistent with a broader increase in publications addressing innate immune signaling, microglia-associated neuroinflammation, and peripheral–central immune crosstalk (35).

### 4.2 Global research landscape reveals complementary national roles and uneven collaboration intensity

Country-level collaboration analysis demonstrated pronounced heterogeneity in research productivity, citation impact, and international collaboration patterns. China, the United States, and India emerged as leading contributors, reflecting differences in experimental capacity, research focus, and collaborative networks. Smaller or emerging research-active countries showed limited connectivity. These patterns indicate diverse national contributions to the field and suggest opportunities for enhanced cross-regional collaboration to harmonize research approaches. From an immunological standpoint, such fragmentation may hinder the harmonization of immune-related outcome measures, including inflammatory biomarkers, microglial activation states, and cytokine profiling relevant to depressive phenotypes.

### 4.3 Fragmented author and institutional networks reflect thematic specialization and disciplinary compartmentalization

Core author and institutional mapping revealed a relatively stable yet fragmented research community. Despite a substantial pool of core contributors, collaborative networks remain sparse and localized. These author clusters largely reflect narrow technical specializations—predominantly brain-targeted delivery, nanoparticle-based antidepressants, and intranasal strategies—indicating that the field’s momentum is currently driven by formulation-focused research. At the institutional level, regional hubs in Asia and the Middle East have pioneered methodological innovations through their expertise in pharmaceutical sciences, but they appear to operate relatively independently from complementary fields such as immunology, neuroscience, and psychiatry. Bridging these disciplines may foster more integrative research in the future, but this remains a developing trend rather than an established mechanism.

### 4.4 Intellectual foundations emphasize nanotechnology-enabled delivery and analytical innovation

Highly cited references illustrate that early studies primarily focused on nanotechnology-based delivery systems, optimization of bioactive compounds, and analytical or sensing methodologies. These foundational contributions shaped subsequent research trajectories, providing methodological and conceptual frameworks. Over time, publications increasingly addressed depression-specific challenges, including blood–brain barrier penetration, pharmacokinetic optimization, and central nervous system targeting. These trends suggest that attention to immune-related aspects in nano-depression research is emerging but still developing.

### 4.5 Hotspots and development trends

#### 4.5.1 Nanotechnology in modulating neuroinflammation and immune dysregulation in depression

Accumulating evidence suggests that depression is not solely a neurotransmitter disorder but is closely associated with chronic low-grade inflammation, immune imbalance, and oxidative stress (36–38). Consistent with this trend, keyword co-occurrence and burst analyses in our study showed that terms such as inflammation, oxidative stress, microglia, and cytokines have become more prominent in recent years, suggesting increasing research attention to immune-related aspects of nano–depression research. Within this context, nanotechnology has been increasingly discussed in relation to its potential relevance to drug delivery, surface modification, and bioavailability. The literature retrieved in this study also indicates growing interest in nanoparticle-based systems for the delivery of anti-inflammatory, antioxidant, and immunomodulatory compounds, particularly in studies addressing blood–brain barrier transport and neuroimmune-related targets in the central nervous system. Through these mechanisms, nanomaterials can attenuate excessive pro-inflammatory cytokine release (e.g., TNF-α, IL-1β, and IL-6), suppress microglial overactivation, and mitigate oxidative stress–induced neuronal damage (39).

In addition, emerging evidence suggests that certain nanomaterials themselves may exert intrinsic immunomodulatory effects by influencing redox homeostasis, mitochondrial function, and inflammasome activation (40–42). The convergence of nanotechnology with neuroimmunology highlights a conceptual transition from symptom-oriented antidepressant strategies toward mechanism-driven interventions targeting immune dysregulation and neuroinflammation in depression.

Importantly, the increasing prominence of immune-related themes in our bibliometric maps suggests growing scholarly attention to the heterogeneity of depressive disorders and the possible involvement of immune-related processes in specific patient subgroups. In this context, the literature has shown emerging interest in more individualized immune-oriented research perspectives, including studies that consider inflammatory biomarkers and related immune signatures. Our findings further indicate that nanotechnology is increasingly discussed in connection with diagnostic and therapeutic applications relevant to immune-associated depression research, such as biosensing platforms, biomarker detection, and targeted delivery strategies. Taken together, these patterns reflect an expanding research interest in the potential intersection of immune stratification, nanodiagnostic tools, and nanotechnology-based intervention approaches in depression research.

#### 4.5.2 Nano-enabled therapeutic and diagnostic strategies for depressive disorders

Beyond mechanistic exploration, nano-enabled therapeutic and diagnostic applications constitute another major research hotspot. Co-citation clustering identified prominent themes related to nanoparticle-based drug delivery systems, extracellular vesicles, and biosensing technologies, reflecting the expanding translational scope of the field. Nanotechnology-based drug delivery platforms have been extensively investigated to improve the pharmacokinetic profiles and therapeutic efficacy of conventional antidepressants, natural compounds, and emerging immunomodulatory agents. By enhancing brain targeting, prolonging circulation time, and enabling controlled release, nanocarriers may reduce systemic side effects while maximizing therapeutic outcomes. Intranasal nano-delivery strategies, in particular, have attracted increasing attention due to their non-invasive nature and ability to bypass the blood–brain barrier.

In parallel, extracellular vesicles and biomimetic nanocarriers have emerged as promising tools for precision therapy, owing to their low immunogenicity and inherent ability to participate in intercellular communication (43–45). Moreover, the growing prominence of biosensor-related keywords underscores rising interest in nano-enabled diagnostic approaches, including the detection of inflammatory biomarkers, oxidative stress indicators, and immune-related molecular signatures associated with depression. Such nano-enabled diagnostic approaches may facilitate immune stratification of depressive patients based on inflammatory or oxidative stress signatures, supporting personalized immunomodulatory interventions. These advances suggest a gradual shift toward integrated diagnostic–therapeutic (theranostic) frameworks that align with the principles of precision medicine.

#### 4.5.3 Safety considerations and translational challenges of nanotechnology-based interventions

While the therapeutic and diagnostic potential of nanotechnology in depression is compelling, its clinical translation hinges on addressing significant biosafety bottlenecks. Our analysis of keyword trends and citation bursts underscores a persistent focus on toxicity and biocompatibility—a reflection of the growing need for safety protocols that align with the long-term nature of psychiatric care. Unlike acute interventions, antidepressants typically involve months or years of administration; thus, safety evaluations must extend beyond simple cytotoxicity to account for chronic CNS exposure. Potential risks, such as nanomaterial accumulation in the brain and reticuloendothelial system, material-specific neurotoxicity, or subtle shifts in neuroimmune homeostasis, require rigorous investigation. Moreover, the current lack of consistency in nanomaterial characterization and dosing regimens remains a hurdle for reproducing preclinical findings in a clinical setting.

The clinical reality of maintenance therapy necessitates a shift from short-term exposure studies to chronic, repeated-dosing paradigms. We argue that longitudinal assessments should prioritize not only clearance kinetics and biodistribution but also integrated readouts of neurobehavioral health and neuroimmune status (e.g., microglial priming and cytokine profiles). Leveraging dose-accumulation modeling and cerebrospinal fluid assays could offer a more predictive view of how these materials behave over time—a perspective that is uniquely critical for long-term psychiatric management.

The interaction between nanomaterials and the blood–brain barrier (BBB) represents another pivotal, yet complex, concern. Depending on their physicochemical properties, repeated exposure to nanoparticles might insidiously alter endothelial function or tight-junction integrity. This poses a double-edged risk: while it might facilitate drug entry, a compromised BBB could also permit the “leakage” of peripheral inflammatory signals into the CNS, potentially amplifying neuroinflammatory processes. Consequently, evaluating BBB integrity and “neuroinflammatory liability” (e.g., inflammasome activation) should be viewed as a non-negotiable component of any translational safety package.

## 5 Limitations

This study has several limitations that should be acknowledged. First, the analysis was based on publications retrieved from the Web of Science Core Collection and Scopus databases. Although these databases provide comprehensive bibliographic records, relevant studies indexed in other sources such as Embase, PubMed Central, and regional databases may not have been fully captured. Second, bibliometric analyses are influenced by database indexing practices and parameter settings, which may overrepresent highly cited authors, institutions, or journals. Third, clustering based on keyword co-occurrence and co-citation patterns provides a macro level structural map but cannot directly evaluate study quality, experimental rigor, or clinical effect sizes.

Furthermore, certain thematic imbalances within the psychiatry and immunology interface must be acknowledged. The current literature remains heavily skewed toward preclinical systems, with a predominant focus on stress based rodent models and *in vitro* assays, whereas granular immunophenotypic data from clinical cohorts, including longitudinal cerebrospinal fluid or peripheral blood profiling, remain comparatively scarce. This gap may limit the direct translational relevance of the identified hotspots. Additionally, bibliometric mapping, by its nature as a lagging indicator, may underrepresent nascent but important frontiers such as the gut microbiome, immunity, and nanotechnology axis, as well as exosome mediated immunomodulation, which have not yet reached a publication tipping point. Crucially, we emphasize that citation based metrics reflect academic visibility and scholarly discourse rather than clinical therapeutic efficacy. Consequently, the trends identified in this study should be regarded as hypothesis generating frameworks for future research rather than confirmatory evidence of clinical success.

## 6 Conclusion

This bibliometric analysis provides a comprehensive overview of the evolving research landscape at the intersection of nanotechnology and depression. The findings demonstrate sustained and accelerating growth in publication output, the emergence of a relatively stable yet fragmented research community, and a clear thematic evolution from neurotransmitter-centered studies toward brain-targeted delivery systems and mechanism-oriented investigations involving oxidative stress and neuroinflammation. China, the United States, and India have played complementary roles in shaping the field through research productivity, citation impact, and international collaboration. Highly cited studies have established strong methodological and conceptual foundations, while emerging themes highlight increasing integration of nanotechnology with contemporary neurobiological models of depression. Despite these advances, clinical translation of nano-enabled therapeutic strategies remains limited. Future research integrating nanomaterial innovation with mechanistic insight, standardized evaluation, and well-designed clinical studies will be essential to advance nano-based approaches toward effective and clinically applicable interventions for depression. Based on the bibliometric signals identified here, we propose the following actionable research priorities. (1) Validation of Immune Stratification via Multicenter Cohorts: Efforts should prioritize prospective clinical studies to validate immunologically defined depression subtypes. These cohorts will be essential to test whether nano-enabled diagnostic platforms can accurately stratify patients and monitor longitudinal treatment response in real-world settings. (2) Next-Generation Diagnostic Precision: Research must accelerate the development of point-of-care biosensors and advanced imaging probes. These tools should target oxidative-stress endophenotypes, enabling clinicians to track neuroimmune dynamics with high spatial and temporal resolution. (3) Targeted Translation for Treatment-Resistant Populations: Translational efforts should be concentrated on biomarker-guided nanotherapeutics—such as microglia-targeted or stimuli-responsive platforms—specifically tailored for inflammatory depression subtypes that remain refractory to conventional pharmacotherapy. (4) Infrastructural Synergy and Data Harmonization: The establishment of open-access repositories and international consortia is imperative. Such collaborative frameworks will harmonize nanomaterial characterization and immune endpoints, ensuring that preclinical findings are reproducible and cross-comparable. (5) Regulatory-Grade Safety Pipelines for Chronic Use: Future studies must implement safety assessments that mirror the clinical reality of long-term psychiatric management. This includes standardized protocols for evaluating cumulative CNS exposure, blood–brain barrier integrity, and neuroinflammatory liability under repeated-dosing paradigms.

## Data Availability

The original contributions presented in the study are included in the article/**Supplementary Material**. Further inquiries can be directed to the corresponding author.
