# Supplementary material for: Immunological landscape of nanotechnology-based depression research: a bibliometric analysis of neuroinflammation and immune modulation
Source: Front Immunol. 2026 May 1;17:1791933. doi: 10.3389/fimmu.2026.1791933 (PMC13175822; doi:10.3389/fimmu.2026.1791933)
Supplement: Supplementary file 1 [file Table1.docx]

| Web of Science Core Collection | #1 TS=(depression) OR TS=(Major Depressive Disorder) OR TS=(Persistent Depressive Disorder) OR TS=(Seasonal Affective Disorder) OR TS=(Postnatal Depression) | 816194 |
| --- | --- | --- |
|  | #2 TS=nano* (Topic) | 317651 |
|  | #3 #1 AND #2 | 3679 |
|  | #4 Document Type=Article or Review | 3570 |
| Pubmed | #1 ("gut-brain axis" OR "microbiota-gut-brain axis" OR "microbiome-gut-brain axis" OR "brain-gut axis" OR "gut-brain communication") | 10189 |
|  | #2 ("neurotransmitter*" OR "serotonin" OR "5-HT" OR "dopamine" OR "GABA" OR "glutamate" OR "norepinephrine" OR "noradrenaline" OR "acetylcholine" OR "histamine"OR"neurotransmitter precursor" OR "tryptophan" OR "tyrosine" OR "glutamic acid"OR "choline" OR "dopa precursor") | 1,194,852 |
|  | #3 #1AND#2 | 2300 |
|  | #4 Language=English | 2263 |
|  | #5 Document Type=Article or Review | 2225 |
|  | #6 #1 AND #2 AND #3 AND #4 AND #5 | 2225 |
